# Exploring the use of virtual reality to manage distress in adolescent patients in emergency departments: A feasibility study

**DOI:** 10.1111/1742-6723.13945

**Published:** 2022-03-02

**Authors:** Elham Zolfaghari, Brad Ridout, Sharon Medlow, Andrew Campbell, Andrew Coggins, Margaret Murphy, Shefali Jani, Deepali Thosar, Brenda K Wiederhold, Mark Wiederhold, Katharine Steinbeck

**Affiliations:** ^1^ Specialty of Child and Adolescent Health Faculty of Medicine and Health, The University of Sydney Sydney New South Wales Australia; ^2^ Academic Department of Adolescent Medicine The Children's Hospital at Westmead Sydney New South Wales Australia; ^3^ Discipline of Biomedical Informatics and Digital Health Cyberpsychology Research Group, Faculty of Medicine and Health, The University of Sydney Sydney New South Wales Australia; ^4^ Department of Emergency Medicine, Westmead Hospital Western Sydney Local Health District Sydney New South Wales Australia; ^5^ Discipline of Emergency Medicine Sydney Medical School, The University of Sydney Sydney New South Wales Australia; ^6^ Department of Emergency Medicine The Children's Hospital at Westmead Sydney New South Wales Australia; ^7^ Virtual Reality Medical Center Scripps Memorial Hospital La Jolla California USA

**Keywords:** adolescents and young adult, distress, emergency department, virtual reality

## Abstract

**Objective:**

The present study aimed to explore the feasibility and potential benefits of deploying virtual reality (VR) for adolescents in the ED.

**Methods:**

This multi‐centre study was undertaken in paediatric and adult EDs in two university teaching hospitals. Twenty‐six participants who had voluntarily attended the ED received the VR intervention. Pre‐ and post‐measures assessing changes in state anxiety, stress and affect, and physical biomarkers were obtained.

**Results:**

The use of VR intervention was associated with significant reductions in distress (Short State Stress Questionnaire – Distress Subscale; *t* = 4.55, *P* < 0.001) and negative affect (the International Positive and Negative Affect Scale – Short Form version; *t* = 4.99, *P* < 0.001). Most participants chose ‘Netflix’ as their content of choice. The technology was well received by the participants with subjective reports indicating that receiving VR intervention was ‘insanely cool’, ‘takes you away from what's actually happening’ and some participants felt ‘privileged to get this experience in a hospital’.

**Conclusions:**

VR technology can effectively be used in EDs to assist adolescents and young adults better manage their distress and take steps towards activating more self‐control mechanisms that will in turn allow for more meaningful engagements to be established with health clinicians. This technology has broad implications for reducing distress in adolescents in a variety of clinical contexts.

## Abstract 2

Virtual reality technology can effectively be used in EDs to assist adolescents and young adults better manage their distress and take steps towards activating more self‐control mechanisms that will in turn allow for more meaningful engagements to be established with health clinicians. This technology has broad implications for reducing distress in adolescents in a variety of clinical contexts.

**Key findings**

- It is feasible to initiate a VR distraction for adolescents waiting in the ED.
- The VR intervention reduced stress as measured by questionnaire, but not as measured by biomarkers of stress.
- VR scored highly on patient acceptability.

## Introduction

ED presentations for adolescents and young adults (AYAs) have steadily increased over the past decade in Australia and internationally.^1^ Possible drivers include the convenience of ‘opening’ hours, cost and perceptions of safety, anonymity and confidentiality.^1^, ^2^, ^3^ In practice, this means that young people often present to EDs with health issues that could be addressed in community settings, and as a result are triaged as low acuity presentations. This results in longer waiting times in unfamiliar and often stressful environments,^4^ which in turn may increase personal stress and anxiety and make it difficult for young people to regulate their emotions.

Removal from the source of distress, the provision of a quiet and/or private space, distraction tasks (e.g. colouring books)^5^ and quiet music are all simple techniques that may reduce distress in multiple clinical settings.^6^, ^7^ The efficacy of these techniques has been quantified by biomarkers demonstrating a reduction in body movement, and reduced pulse rate, blood pressure (BP) and respiratory rate.^7^ However, these approaches may not always be viable, especially in EDs, where bright lighting, noise and constant movement are part of standard functioning. Concerns have been raised about the suitability of EDs to address the specific needs of AYAs.^8^ AYAs have voiced concerns about the ambience, comfort and extended waiting times in EDs, and stated they would prefer the ED to allow for access to appropriate technology and entertainment.^8^, ^9^, ^10^

Over the past decade, the health sector has shown increasing interest in the use of virtual reality (VR), with studies conducted in a wide range of clinical conditions, including pain management,^11^, ^12^ rehabilitation,^13^ anxiety,^14^ phobias^15^ and post‐traumatic stress disorder.^16^ The mechanisms by which VR reduces the experience of negative sensations such as anxiety or distress have mostly been attributed to active distraction and potential immersion in an alternative environment.^12^ Specifically, VR interventions engage cognitive resources through high multisensory stimulation (immersion in a three‐dimensional spatial environment). This produces positive responses in the autonomic nervous system, in real time.^17^, ^18^, ^19^ Previously, it has been demonstrated that active distraction using VR significantly reduces levels of anxiety and anger in adult patients in the ED.^20^

While AYAs are increasingly using VR technology,^21^ it should not be assumed that VR will be a successful intervention in the ED setting. To our knowledge, no study has investigated the use of VR to contain anxiety and distressing emotions in AYAs in EDs.^22^ Importantly, no studies have incorporated stakeholder (especially young persons) input into the design, delivery and evaluation of this novel research.

The present study aims to explore the potential benefits of deploying VR in the ED environment. The primary objective was to ascertain the feasibility and practicality of a brief VR intervention in the ED setting. The secondary objective was to determine whether the intervention was associated with reduced signs and symptoms of anxiety and distress.

## Methods

### Input from young people

The Wellbeing Health & Youth (WH&Y) Commission is part of the senior author's Australian National Health and Medical Research Council Centre of Research Excellence in Adolescent Health (http://why.org.au/). Seventeen young people from the WH&Y Commission reviewed the present study's research protocol, considered ethical issues arising and provided input into the proposed methods.

### Settings

The present study was conducted in co‐located, but totally separate paediatric and adult EDs with different patient cohorts in two university teaching hospitals in Westmead, Western Sydney, Australia.

### Eligibility

Participant inclusion criteria were: patients who were ambulatory, between the ages of 14 and 20 years (which includes adolescence 10–19 years and young adulthood 18–25 years) and triaged as category 3, 4 or 5. The Australasian Triage Scale (ATS), developed by the Australasian College for Emergency Medicine, is a standardised clinical tool used to establish the maximum waiting time, based on clinical urgency, for medical assessment and treatment of a patient.^23^ Categories 3, 4 and 5 indicate that 75%, 70% and 70% of patients should ideally be seen in 30, 60 and 120 min, respectively. Exclusion criteria were: overt psychosis, alcohol and/or other substance intoxication, history of epilepsy or severe headaches (as these are recognised side‐effects of VR in some people) or incapacity to follow instructions for VR use or complete questionnaires.

### Procedure

ED patients identified by triage staff as meeting the inclusion criteria were approached by a research assistant in the waiting area of the ED for recruitment. Informed, written consent was obtained from each participant and where relevant from their parent/guardian. Each participant was taken to a quiet space within the department and received the VR intervention for between 20 and 40 min. Participants were able to choose from select pre‐loaded apps: Netflix TV Shows with a G or PG rating (e.g. Big Bang Theory, Glee), a mindfulness app (TRIPP) and a non‐violent game (Tetris).

### Measures

Pre‐ and post‐intervention measures included questionnaires and biomarkers.

#### Questionnaires

Three widely used and well‐validated questionnaires were administered to all participants before and after the VR experience to assess changes in anxiety, stress and affect. These were: the six‐item short‐form of the state scale of the Spielberger State–Trait Anxiety Inventory (STAI‐6);^24^ the Short State Stress Questionnaire – Distress Subscale (SSSQ‐D, eight items);^25^ and the International Positive and Negative Affect Scale – Short Form version (I‐PANAS‐SF, 10 items).^26^ Post‐intervention measures were followed by a brief user experience interview consisting of Yes/No and open response questions, including: a general ‘think aloud’ probe (i.e. ‘when you think about your experience using the VR device, what's the first thing that comes to your mind?’); feedback on using the VR device (level of comfort, any concerns); rating the VR experience (favourite and least favourite part, duration); and insights on perceived effects (on levels of relaxation, anxiety/concern, pain).

#### Physical biomarkers

A pulse oximeter recorded heart rate to assess for tachycardia, and BP was measured using an automated cuff. Readings were taken pre‐ and post‐intervention to evaluate physical responses to VR.

#### Power calculations and statistical analysis

This was an exploratory study and recruitment was limited by COVID‐19. Data were collected intermittently between November 2020 and February 2021, which reduced the proposed number of participants by 20%. AYAs attending the adult hospital at Westmead were more inclined to participate (*n* = 23; 80%). Outcomes for the pre‐ and post‐intervention questionnaires and biomarker measures were compared within participants using paired sample *t* tests.

Responses to the open questions were analysed using simple content analysis. Each response was read and assigned an open code that summarised the key idea in the response.^27^, ^28^ Similar codes were grouped under themes. BR reviewed all coding and ACampbell provided a second review, with any disagreements resolved by discussion.

### Ethics

The present study was approved by the Sydney Children's Hospital Network Ethics and Governance committees (2020/ETH00646) and the Westmead Hospital Ethics and Governance committees (ref. no. 6584‐2020/STE02003), Sydney, New South Wales, Australia.

## Results

### Sample characteristics

A total of 104 potential participants were approached, with 32 individuals consenting to participate. The reasons for not consenting included: not interested (*n* = 34), medical/mental health contraindication (*n* = 17), parent not consenting (*n* = 4) and other (*n* = 17). Although data on how long individuals had been waiting in emergency was not recorded, the research assistant noted that recruitment of participants appeared to be more successful after they had waited for some time without being seen (generally an hour), rather than approaching straight after triage, when they were more likely to decline to participate. Participants included 32 (19 women) young people (mean age 16.8, standard deviation [SD] 1.91; range 16–20 years) who voluntarily attended the EDs. Common reasons for presentation to the ED were injury (*n* = 9), abdominal pain (*n* = 6), mental health (*n* = 4), headache (*n* = 2), with no commonality among the remaining presentations. Six participants did not complete the study as a result of being called in for tests/treatment, and one experienced a panic attack before beginning the VR intervention. One participant felt dizzy, but completed the study. This resulted in 26 usable cases for the study.

### Physiological and psychological measures

Mean pre‐ and post‐intervention scores on physiological and psychological measures are presented in Table 1. All participants included in the study completed all quantitative measures, with the exception of one participant for whom BP readings were not recorded. No significant differences were detected for any of the physical biomarkers measured. However, there was a significant reduction in distress as measured by the SSSQ‐D, from 13.1 (SD 5.5) pre‐intervention to 9.4 (SD 3.1) post‐intervention (*t* = 4.55, *P* < 0.001; *d* = 0.84), and a significant reduction in negative affect as measured by the I‐PANAS‐SF, from 9.3 (SD 3.9) pre‐intervention to 6.7 (SD 2.5) post‐intervention (*t* = 4.99, *P* < 0.001; *d* = 0.81). Both of these reductions are clinically significant, and the effect size for each was found to exceed Cohen's convention for a large statistical effect (*d* > 0.80).^29^ There were no significant differences in pre‐ and post‐intervention measures of anxiety or positive affect (as measured by the I‐PANAP‐SF).

**TABLE 1:** Mean pre‐ and post‐intervention scores on physiological and psychological measures

Measure	Pre‐intervention		Post‐intervention		Difference	df	*t*	*P*‐value	Cohen's *d*
	Mean	SD	Mean	SD					
Pulse	87	12.7	85	15.4	2.8	25	1.68	0.105	0.2
BP – systolic	116	10.3	116	11.5	−0.1	24	−0.07	0.944	0.01
BP – diastolic	75	9.5	77	7.4	−1.4	24	−0.73	0.475	0.17
Oxygen saturation	98	1.1	98	1.9	0.4	25	0.87	0.390	0.21
State anxiety (STAI‐S‐F)	12.4	2.4	12.4	1.7	0.4	25	0.07	0.942	0.02
Distress (SSSQ‐D)	13.1	5.5	9.4	3.1	3.7	25	4.55	0.000*	0.84
Positive affect (I‐PANAS‐SF)	11.4	3.5	12.2	4.8	−0.9	25	−1.04	0.309	0.21
Negative affect (I‐PANAS‐SF)	9.3	3.9	6.7	2.5	2.7	25	4.99	0.000*	0.81

* *P* < 0.001.

BP, blood pressure; I‐PANAS‐SF, International Positive and Negative Affect Scale; SD, standard deviation; SSSQ‐D, Short State Stress Questionnaire – Distress Subscale; STAI‐S‐SF, Spielberger State−Trait Anxiety Inventory – State Subscale, Short form version.

### Participant experience

All but one participant said they found wearing the device comfortable, with one reporting that it made their eyes ‘blurry and sore’. This participant also reported feeling a little dizzy during the VR experience. Two participants noted that the device was heavy. All participants said that they had enough space while using the device, and most thought that the duration of the VR experience was about right, with three saying that it was too short and only one saying it was too long.

Twelve participants chose ‘Netflix’ as their content choice, 11 chose Tetris and one participant tried both. Just two participants chose the ‘Mindfulness’ option, and both mentioned doing so only because there were technical difficulties with ‘Netflix’ at the time. Most participants (80%) reported being happy with their choice and said they would make the same choice again, while three participants said they were happy but would try something else next time. Two participants said that they found the content choices limited.

When asked whether the VR experience had any impact on the pain or discomfort they had when they entered the ED, responses were relatively evenly split, with 46% saying that they did experience a reduction while the rest experienced no change.

### Participant qualitative feedback

Table 2 presents the responses to qualitative questions and demonstrates that these were positive. Of note, when asked how the VR experience made their body feel, the majority (73%) said ‘calm’ or ‘relaxed’. When asked how the VR experience made their mind feel, the most common response was also ‘calm’ or ‘relaxed’ (62%). When asked why they thought the VR experience made their body and mind feel this way, most responses related to distraction (50%) or immersion (39%).

**TABLE 2:** Common themes in responses to open questions

Question	Theme	Frequency†	Percent
‘When you think about your experience using the VR device, what is the first thing that comes to your mind?’	Fun experience	11	42
	Amazing/novel experience	8	39
	Good distraction	5	19
	Calming	2	8
‘If a friend or family member asked you about the device, what would you tell them about it?’	Recommend it	15	5
	Amazing/novel experience	4	15
	Fun experience	3	12
	Good distraction	2	8
	Calming	1	4
	Immersive	1	4
‘What was it like to have this type of immersive experience in this setting?’	Good distraction	11	42
	Calming	6	23
	Weird	6	23
	Forget that in hospital	5	19
	Fun	4	15
‘What was your favourite part about your VR experience?’	Realistic three‐dimensional imagery	7	27
	Content	6	23
	Immersion	5	19
	Calming	3	12
	Good distraction	2	8
	Learning new technology	1	4
	Everything	1	4
‘What was your least favourite part about your VR experience?’	Nothing	11	42
	Blurry/itchy eyes	3	12
	Device heavy	2	8
	Feeling dizzy	2	8
	Duration too long	1	4
	Volume too quiet	1	4
	Marks on face afterwards	1	4
	Feet felt cold	1	4
‘How did participating in this study make your body feel?’	Calm/relaxed	19	73
	Distracted from pain	2	8
	No change	2	8
	Good	1	4
	More active (playing game)	1	4
	Dizzy	1	4
‘How did participating in this study make your mind feel?’	Calm/relaxed	16	62
	Sense of being somewhere else	3	12
	Focused (on playing game)	3	12
	Good	2	8
	No change	1	4
‘Why do you think the VR experience made your body and mind feel this way?’	Good distraction	13	50
	Immersion in a different world	10	39
	Comforting	2	8
	Relaxing	1	4
	Novelty	1	4
	Concentration required for game	1	4

† Some frequencies total more than 100% because of multiple themes identified in some responses.

### Staff qualitative feedback

The original protocol included obtaining feedback from any ED staff who had direct care contact with participants about their own perceptions of the VR trial. Unfortunately, this proved impossible to capture given the time pressures on staff in a busy ED setting, particularly in the context of COVID‐19 protocols.

## Discussion

The use of VR in the health sector is growing as VR headsets become increasingly more affordable, lightweight and therefore practical. However, there is still untapped potential for use among adolescents in hospital settings.^30^ Using both quantitative and qualitative measures, the present study has provided a better understanding of how best to incorporate VR technology as an effective and feasible tool to manage anxiety and distress in AYA patients attending the ED. To the best of our knowledge, this is the first study to assess the feasibility and potential benefits of VR technology in ED with an AYA population, and to incorporate young people into the design, delivery and evaluation of it.

Our qualitative data present overwhelmingly positive feedback from participants on using VR while in the ED waiting room, with many noting how much fun and/or novel the experience was. While 54% said that using VR did not have any impact on their pain or discomfort, most participants said that it did make them feel calmer and/or relaxed. This was reflected in the significant reduction in short state distress reported by participants, as well as the significant reduction in negative affect.

While these effects have been reported in various other research in the field with older populations,^30^ it is of interest that digitally sophisticated AYAs had a similar response. The positive responses from participants, including the ability of VR to distract and immerse, even in busy clinical settings, presents ‘proof of concept’ and encourages further use of this technology in EDs.

We were unable to demonstrate any significant changes in physiological markers of distress or anxiety. However, it should be noted that mean pre‐intervention scores on these measures were within the normal range, and therefore may not have been high enough to allow for meaningful reductions to be observed. Further studies are therefore needed to investigate whether VR can effectively reduce physiological markers of distress in AYAs in ED settings, perhaps by including patients experiencing acute pain or undergoing painful interventions.

Staff feedback information was not able to be captured because of the pressures of clinical care in busy EDs (and the COVID‐19 operations pressures experienced during the period of the present study). With this, it is important for further large‐scale studies to capture the impressions of this group of professionals, with consideration to the pragmatics of how qualitative data can be collected without adding workload pressure. However, in order to provide a contemporary, best‐practice model of care for AYAs presenting to EDs, the importance of digital health training needs to be carefully communicated prior to any large‐scale study to outline the potential benefits it may have towards workload and patient management with ED doctors and nurses. Without their support and cooperation, as well as qualitative input by ED staff, the use of VR may be viewed by hospital professionals as counterproductive to their primary healthcare duties, despite patient evidence that it may reduce distress.

Other limitations of the present study were small numbers and a lack of resources to cover 24/7 h in the ED, where there might have been a broader range of presentations. In addition, we did not capture as broad an age range as we wished, with low paediatric ED participant numbers. The latter might potentially be explained by parents being less willing to consent to their child's participation or that the young person was assessed more quickly. Both options could be explored in future studies.

## Conclusions

VR is an innovative and increasingly cost‐effective e‐technology approach to creating a positive feedback loop that allows patients to control their hyper‐emotionality and take steps towards activating more effective self‐control mechanisms. This, in turn, should allow for more meaningful engagements to be established with health clinicians. We have shown for the first time that using VR in the ED is feasible from an AYA patient perspective, and that it supplements existing measures such as free Wi‐Fi to support patients' own devices. It will be important to understand how ED clinicians consider this type of intervention would work if they were responsible for VR interventions with an already busy workload. Our feasibility data suggest these questions are worth pursuing, with broad implications for reducing distress in AYAs and for creating more congenial atmospheres in healthcare waiting areas.

## Data Availability

Data available on request from the authors.
